# Automated classification of osteoporotic vertebral compression fractures in DR images based on improved YOLO26n-seg

**DOI:** 10.3389/fmed.2026.1844808

**Published:** 2026-08-07

**Authors:** Zhixuan Wang, Haowen Lu, Heting Xiao, Xuwei Ling, Yu Chen, Zheming Shen, Minbo Jian, Lingfeng Huang, Quan Zhou, Peng Yang, Tao Liu, Yusen Qiao

**Affiliations:** Department of Orthopaedics, The First Affiliated Hospital of Soochow University, Suzhou, China

**Keywords:** automated classification, deep learning, osteoporotic vertebral compression fracture, SAConv module, YOLO26n-seg

## Abstract

**Background:**

Osteoporotic vertebral compression fractures (OVCFs) are prevalent fragility fractures among the middle-aged and elderly populations. The widely used manual Genant classification for OVCF assessment is limited by low efficiency, high subjectivity, and a significant rate of underreporting on imaging. Furthermore, dedicated deep learning models for accurate classification remain scarce.

**Methods:**

To address these clinical challenges, we proposed an improved YOLO26n-seg-SAConv model by substituting the traditional convolutional downsampling module of YOLO26n-seg with a Switchable Atrous Convolution (SAConv) module. A retrospective dataset comprising 483 digital radiography (DR) images of OVCFs was collected from the First Affiliated Hospital of Soochow University and its affiliated institutions between January 2021 and January 2026. After preprocessing, the data were divided into training and test sets at an 8:2 ratio and augmented with Gaussian noise.

**Results:**

The improved model achieved a mean average precision at an Intersection over Union of 0.5 (mAP50) of 74.2%, representing a 3.9% improvement over the original model. Concurrently, the computational complexity (floating-point operations per second, FLOPs) was reduced by 16.67%. Notably, the modified model demonstrated significantly enhanced classification performance for Type 1 and Type 3 fractures, which conventionally suffer from smaller sample sizes.

**Conclusion:**

The proposed lightweight YOLO26n-seg-SAConv model is suitable for edge deployment. This methodological study verifies the feasibility of automated Genant classification of OVCFs on DR images, laying a technical basis for its further development into an auxiliary triage tool in subsequent clinical translation research.

## 1 Introduction

Osteoporotic vertebral compression fracture (OVCF) is a type of fragility fracture caused by osteoporosis. Due to the decrease in vertebral bone mineral density, bone quality and bone strength, it can occur under mild or even no obvious external force, with thoracolumbar back pain as the core clinical manifestation, which seriously threatens the physical health and quality of life of the middle-aged and elderly (1–3). Studies have shown that osteoporosis causes approximately 8.9 million fractures every year, with one fracture occurring every 3 s on average. The incidence of osteoporotic fractures in women and men over 50 years old reaches 1/3 and 1/5 respectively, and the vertebral body is a high-incidence site for osteoporotic fractures, making OVCF highly prevalent (4, 5).

Current clinical diagnosis and treatment of OVCF rely on the comprehensive judgment of medical history collection, physical examination and imaging examinations, and the selection of treatment plans is also based on the severity of OVCF (6–8). Therefore, imaging examination is a very important part, but imaging underreporting still exists in the clinical diagnosis and treatment process (9–11). Hence, it is essential to adopt standardized and automated classification methods.

At present, deep learning research on OVCF mainly focuses on the early diagnosis of OVCF, that is, identifying whether vertebral fractures occur through deep learning models, and there is a serious lack of research on deep learning models for OVCF classification (12–14). However, OVCF classification can reflect the severity of fractures and has certain guiding significance for clinical diagnosis and treatment (15, 16). There are many classification methods for OVCF at present. The Genant semi-quantitative classification is adopted in this study due to its simple operation, intuitive understanding, and wide application in epidemiological research and clinical diagnosis and treatment (15).

The YOLO series models have been extensively applied across a wide spectrum of medical fields, including radiological diagnosis, stomatology, cytopathology, and histopathology, demonstrating remarkable clinical value and promising translational potential (17–21). Based on the YOLO26n-seg deep learning model, this study constructed an automated OVCF classification system to assist manual diagnosis. According to the Genant classification, the system divides vertebral body conditions into three categories. Through rapid automated classification, it serves as an adjunct to traditional manual classification, helping clinicians improve efficiency and accuracy. It reduces diagnostic and treatment delays caused by underreported imaging findings, provides a reliable basis for clinical treatment planning, and further supports timely clinical decision-making. It also alleviates patients’ pain, lowers the risk of refracture, and ultimately optimizes patient prognosis, offering new technical support for the accurate diagnosis and treatment of OVCF (22).

## 2 Methods

### 2.1 Study subjects

This retrospective study was approved by the Institutional Review Board of the First Affiliated Hospital of Soochow University, and the requirement for informed consent was waived due to its retrospective nature.

A total of 483 lateral DR images of osteoporotic vertebral compression fractures were consecutively collected from our hospital and its affiliated institutions between January 2021 and January 2026. The inclusion criteria were: (1) clinically confirmed OVCF with complete imaging data; (2) clear vertebral morphology on DR images available for Genant classification; (3) age ≥50 years with a diagnosis of primary osteoporosis. The exclusion criteria were: (1) pathological fractures caused by tumors, infections or trauma; (2) severe image artifacts or malposition that affected annotation.

All fractures were classified into Type 1, Type 2, and Type 3 based on the Genant semi-quantitative grading criterion by two senior orthopedic surgeons independently. Inter-rater agreement was quantified using Cohen’s Kappa coefficient, which reached 0.78, reflecting excellent consistency between the two raters. Any discrepant grading results were discussed to reach a consensus after reliability evaluation, and the unified labels were adopted as ground truth for subsequent model analysis. The baseline characteristics of enrolled participants, including age, sex, and fracture grade distribution, are presented in Table 1. The cohort was randomly split into a training set and a test set at an 8:2 ratio. No significant intergroup differences in baseline profiles were observed across Genant subtypes (all *p* > 0.05), demonstrating satisfactory comparability.

**Table 1:** Baseline characteristics of enrolled patients stratified by Genant grade.

Characteristic	Total	Type 1	Type 2	Type 3	*p*-trend
Patients, *n*	483	162	237	84	—
Age, *y*	73.8 ± 8.6	73.6 ± 8.7	73.4 ± 8.7	74.9 ± 8.2	0.3895
Male, *n* (%)	68 (14%)	27 (16.7%)	34 (14.3%)	7 (8.3%)	0.2016
Female, *n* (%)	415 (85.2%)	135 (83.3%)	203 (85.7%)	77(91.7%)	—

Continuous variables (age) were presented as mean ± standard deviation; categorical variables (sex) were expressed as number (percentage). *P*-trend was calculated for linear trend across three Genant subtypes.

### 2.2 Dataset construction

Before inputting the data into the YOLO26 model for training, the DR images underwent a preprocessing stage. This stage included standardizing the images to a unified size and resolution (640 × 640 by default, with the long side set to 640 for rectangular images) and converting them to grayscale. Grayscale conversion reduces the number of image channels and improves the efficiency of image processing.

**Figure 1:**
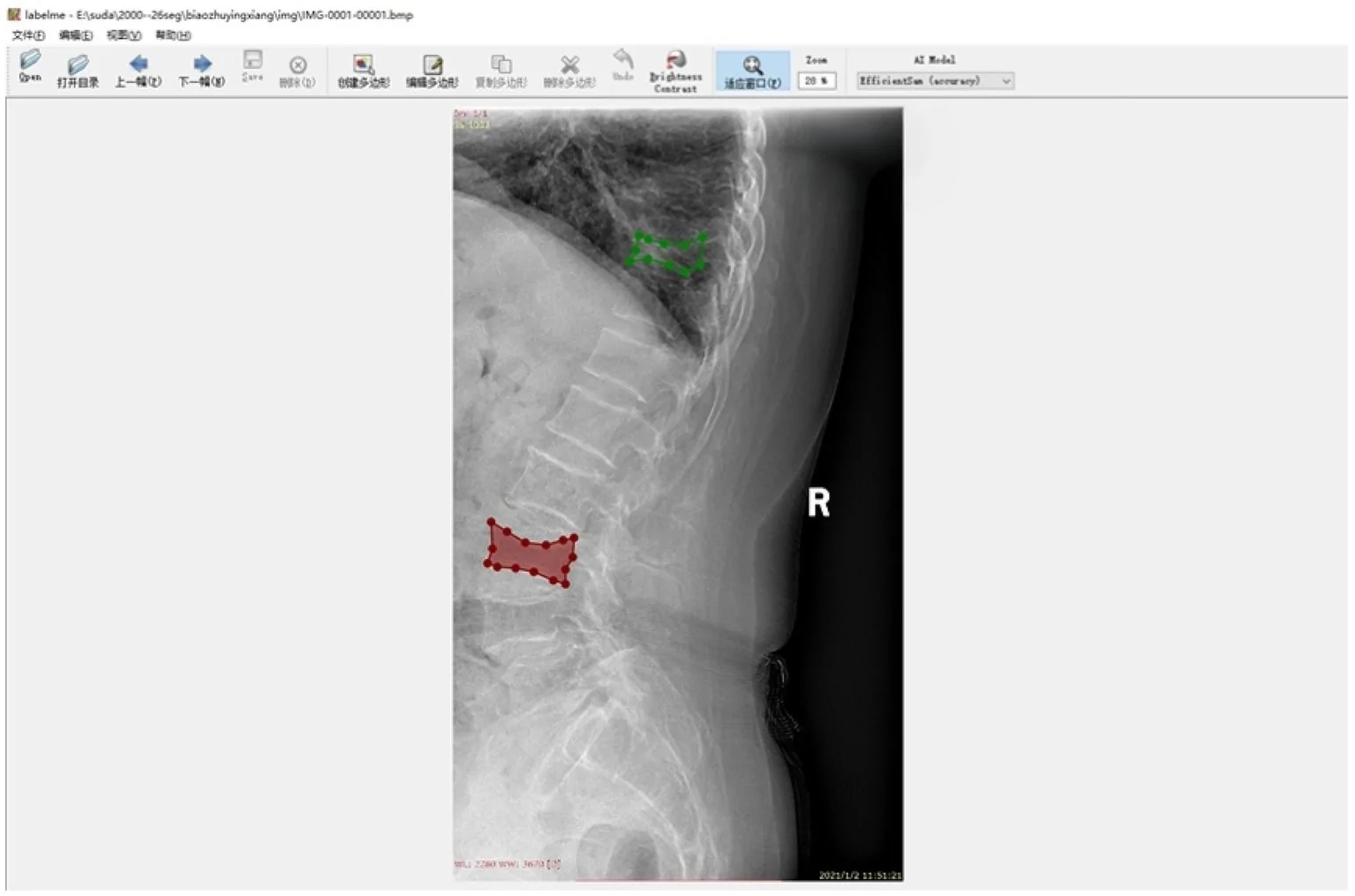
Labelme annotation.

Irregular polygons with different names were used to annotate different types of targets. A specific naming method was adopted in this study: “1” represents Type 1 vertebral compression fracture, “2” represents Type 2 vertebral compression fracture, and “3” represents Type 3 vertebral compression fracture. This naming method is consistently used in the figures of this paper. Finally, a deep learning segmentation and detection dataset for vertebral compression fracture classification was constructed, with the annotation shown in the Figure 1.

**Figure 2:**
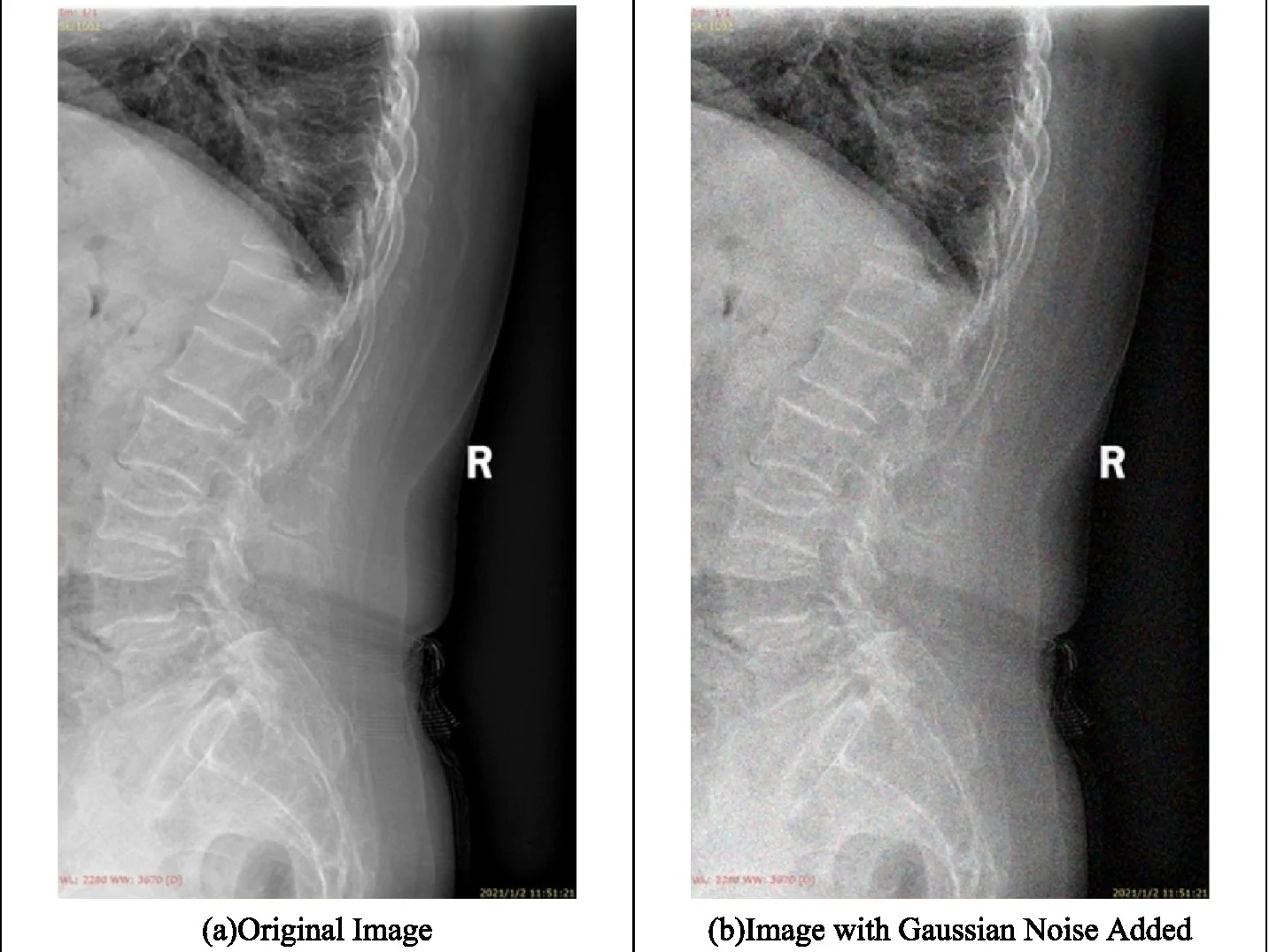
Comparison of image augmentation. In Figure 2, **(a)** shows the original DR image while **(b)** displays the image with added Gaussian noise. It can be observed from the figure that pixel-level Gaussian noise appears in the image. This type of enhancement can improve the model’s robustness under low signal-to-noise ratio conditions.

The 483 DR images selected in this study contained 369 Type 1 targets, 447 Type 2 targets and 100 Type 3 targets in total. The original dataset was first randomly divided into a training set and a test set at an 8:2 ratio by the random seed method. To address the small sample size and improve the robustness of the trained model, Gaussian noise was added to both the training and test sets for data augmentation, thereby enhancing the model’s detection capability under low signal-to-noise ratio conditions and improving model robustness, as shown in the Figure 2.

### 2.3 Principle of YOLO26n-seg algorithm

In January 2026, the Ultralytics research team released YOLO26, the latest iterative version of the YOLO series algorithms. Its structure is basically consistent with the previous generation YOLO11 model, but the model size is greatly reduced by introducing a new calculation method, providing more possibilities for deployment on edge devices. The YOLO26n-seg network consists of a backbone network, a neck network and a head network, and provides versions of n, s, m, l and x with increasing model sizes. To adapt to the deployment of embedded devices, the YOLO26n-seg model was selected in this paper.

As an important evolution of the series, YOLO26 inherits the core architecture of YOLO11 and at the same time carries out systematic optimization and innovation in terms of model accuracy, computational efficiency and deployment flexibility. This algorithm fully considers the adaptability of different computing resources and hardware platforms and exhibits excellent performance in multiple computer vision tasks such as object detection, image segmentation and image classification.

YOLO26 continues to use the C2PSA (Channel-to-Point Spatial Attention) module, which strengthens the attention to key information regions in the image through the spatial attention mechanism, significantly improving the performance of YOLO26 in the feature extraction process and enabling it to more effectively understand information in complex scenes. In addition, YOLO26 also retains the C3K2 module, which combines dilated convolution (C3) and a convolution layer with a convolution kernel size of 2. This design helps to obtain richer feature information at different levels.

In terms of innovation, YOLO26 is a native end-to-end model that directly generates prediction results without non-maximum suppression (NMS). By eliminating this post-processing step, inference becomes faster, lighter and easier to deploy in practical systems.

YOLO26 introduces the MuSGD optimizer, a hybrid of SGD and Muon, which brings enhanced stability and faster convergence, transferring the optimization progress in language models to the field of computer vision.

YOLO26 has introduced targeted improvements for professional tasks, such as semantic segmentation loss and multi-scale prototype modules for the segmentation task. The introduction of semantic segmentation loss improves model convergence, and the upgraded prototype module uses multi-scale information to obtain excellent mask quality. This is of great significance for the segmentation task of vertebral compression fracture classification in DR images in the medical field, and is conducive to improving the detection speed and accuracy of this task.

### 2.4 Improved vertebral compression fracture classification and detection algorithm based on YOLO26n-seg

**Figure 3:**
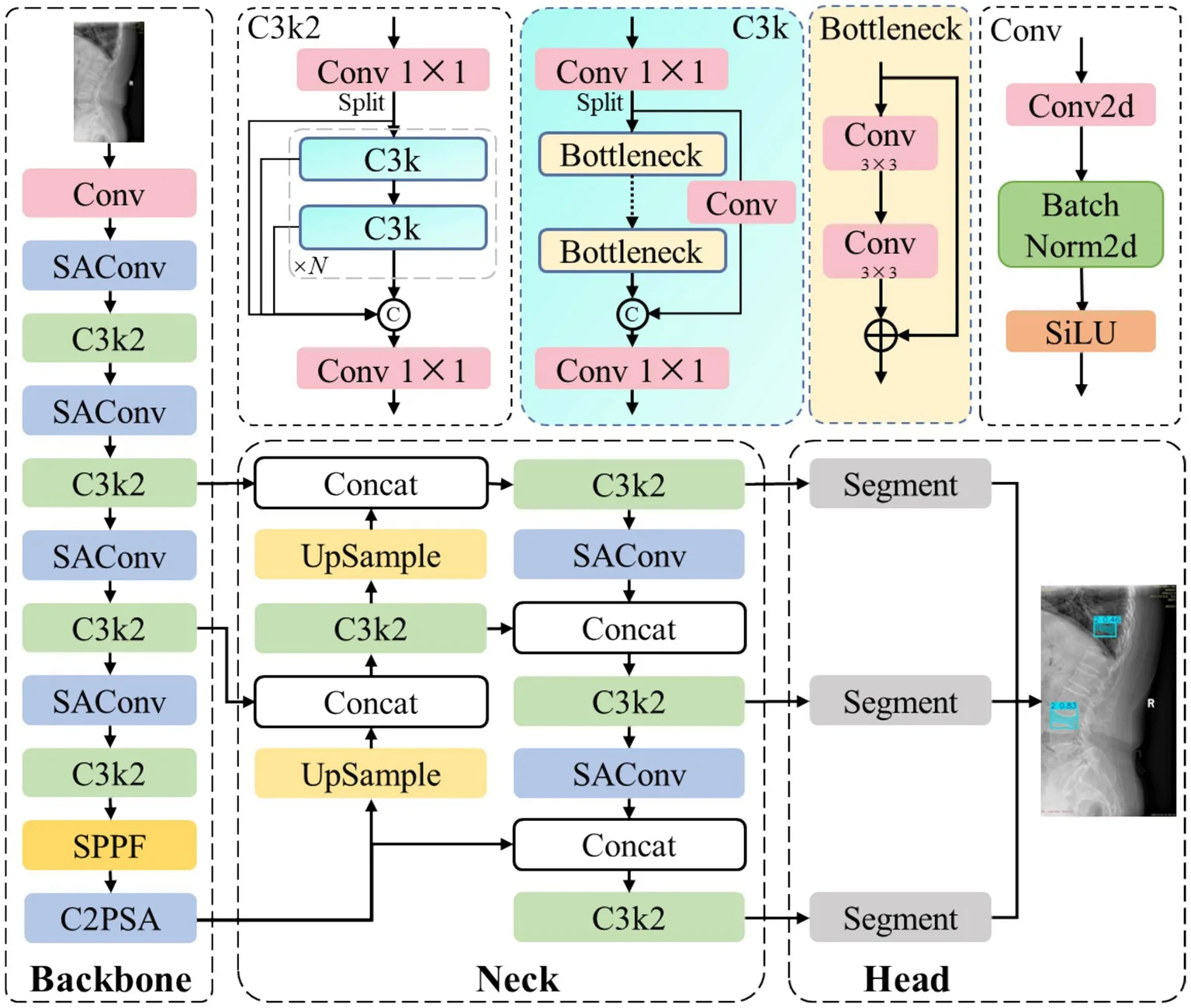
Structure diagram of YOLO26n-seg-SAConv model, Figure 3 illustrates the architecture of the YOLO26n-seg-SAConv model, which is divided into three parts: The backbone network, the neck network, and the detection head. The backbone network consists of Conv, C3k2, SPPF, and C2PSA modules. The neck network is composed of Conv, C3k2 modules, UpSample upsampling modules, and Concat concatenation layers. In the segmentation model, the detection head consists of the Segment module. In the improvement presented in this paper, the Conv modules in both the backbone and neck networks are replaced with SAConv modules, thereby enhancing the detection performance of the model.

To improve the positioning accuracy and specific category judgment accuracy of the model in DR images of vertebral compression fracture classification, reduce the computational complexity and parameters of the model, and improve the overall operation efficiency, key improvements were made on the basis of the YOLO26n-seg model framework to propose the YOLO26n-seg-SAConv model, whose structure is shown in Figure 3. The traditional convolutional downsampling Conv modules in the backbone and neck networks of YOLO26n-seg were replaced with SAConv modules to reduce the model computational complexity, avoid the loss of details in the traditional downsampling process, and improve the model’s feature extraction capability for DR images.

#### 2.4.1 Principle of SAConv module

**Figure 4:**
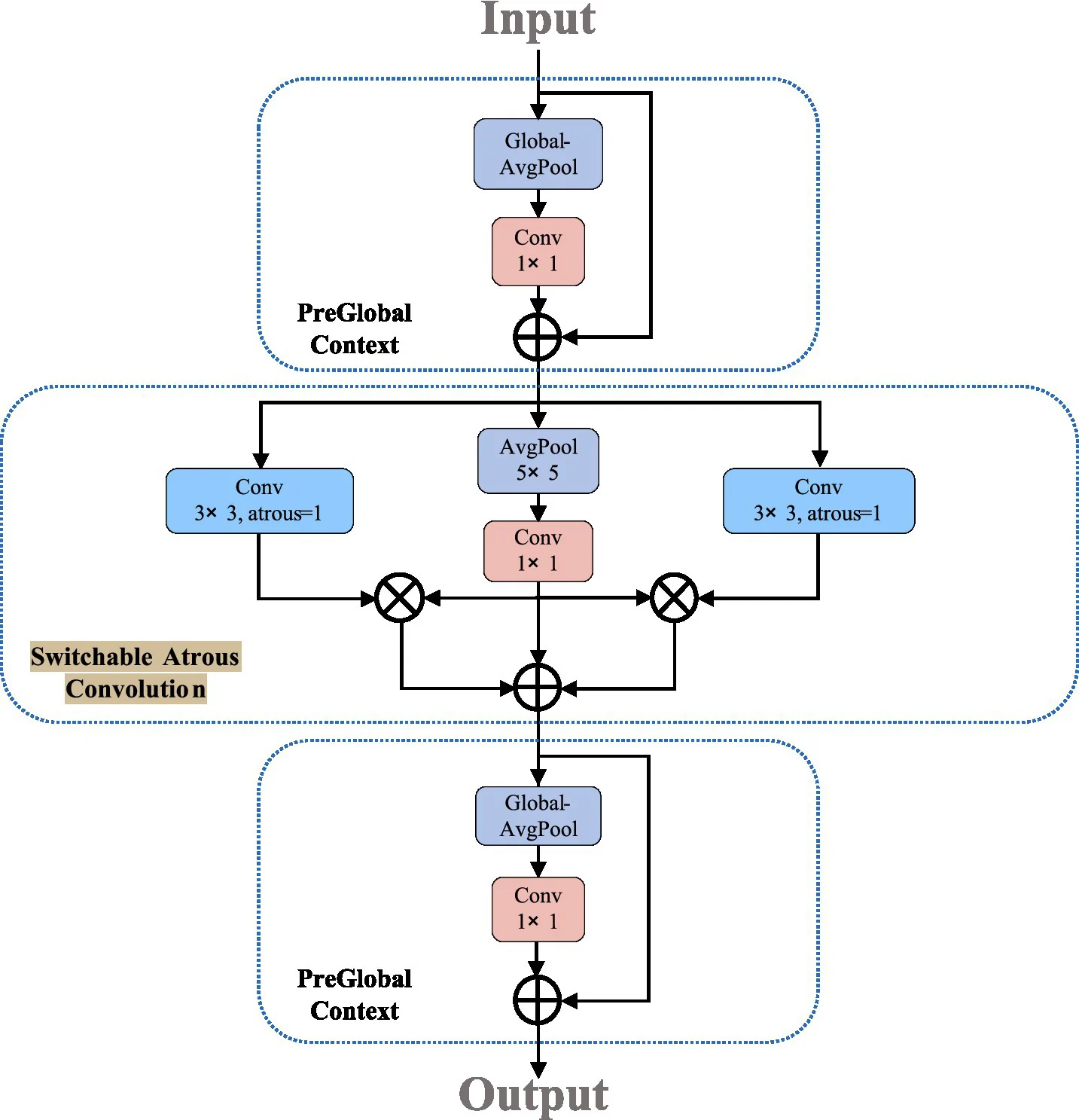
Schematic diagram of SAConv module.

There are small targets in the DR image samples of vertebral compression fracture classification in this paper for target differentiation. The downsampling operation of the traditional Conv module extracts image features through 3 × 3 convolution with a stride of 2, which not only increases the number of model parameters and computational complexity, but also cannot effectively obtain the features of small targets, and is prone to feature loss and missed detection. To solve the problem of information loss of small targets, the SAConv (Switchable Atrous Convolution) module was introduced into the YOLO26n-seg model in this study to replace the Conv downsampling modules in the backbone and neck networks, which effectively solves the problem of information loss of small targets in the feature extraction process. The workflow of the SAConv module is shown in Figure 4.

The SAConv module reconstructs feature extraction information through the combination of dynamic multi-scale perception with different atrous rates and a weight locking mechanism (an adaptive mechanism for features). The module adopts a three-level cascaded architecture: first, the pre-global context module performs spatial semantic modeling on the input features and establishes long-range dependencies to suppress background noise; second, the dual-branch atrous convolution layer implements differentiated sampling strategies in parallel under the constraint of weight sharing—the low atrous rate branch focuses on the fine depiction of local textures, and the high atrous rate branch captures large-scale structural correlations, synchronously decoupling the complex relationship between the target and the environment; a learnable switch function is used, which dynamically evaluates the contribution of dual-path features through a gating mechanism, adaptively fuses them while avoiding grid artifacts caused by high atrous rates, and maximizes parameter efficiency at the same time; finally, the post-global context module recalibrates the fused features and screens out key information with spatial discriminative power. Replacing the Conv module with SAConv in the Backbone combined with context and weight sharing can enhance the model’s generalization ability for targets of different scales. The calculation formula of SAConv is as follows:


$$\begin{array}{l} \text{Output}=S(x)\times \text{Conv}(x,y,1)+(1–S(x))\\ \times \text{Conv}(x,y,\Delta ,y,r) \end{array}$$

Where: Output is the output information; x is the input information; S(x) is the switch function; Conv(x,y,Δ,y,r) is the convolution operation with weight y; *r* is the hyperparameter of the module; Δ y is the trainable value.

### 2.5 Experimental environment and evaluation metrics

#### 2.5.1 Experimental environment

Based on the YOLO26n-seg deep learning network, this study proposed a new YOLO26n-seg-SAConv detection method. The experiment adopted the Ubuntu 20.04 operating system with an NVIDIA 3090 24GB graphics card. Virtual environment configuration: Python version 3.10, PyTorch version 2.1.0, CUDA version 11.8.

The official Ultralytics YOLO26n-seg model (version 8.4.18) was implemented, and image enhancement technologies (e.g., Mosaic, fliplr, scale and translate) were used to improve the robustness of the model. The hyperparameter settings are shown in the Table 2.

**Table 2:** Experimental parameter settings.

Hyperparameter	Value	Hyperparameter	Value
epoch	300	optimizer	SGD
imgsz	640 × 640	Irf	0.01
batch size	16	Ir0	0.01
workers	8	momentum	0.937

#### 2.5.2 Evaluation metrics

In this experiment, precision (P), recall (R) and mean average precision (mAP) were used to evaluate the detection performance of the YOLO26n-seg-SAConv model.

Precision P represents the proportion of actual positive samples among the samples predicted as positive, and its calculation formula is:


$$p=\frac{\mathrm{TP}}{\mathrm{TP}+\mathrm{FP}}$$

Where: TP (True positive) is the number of positive samples correctly classified, that is, the target marked with fractures is detected as fractures; FP (False positive) is the number of negative samples incorrectly detected, that is, the target without marked fractures is detected as fractures.

The calculation formula of recall R is as follows:


$$R=\frac{\mathrm{TP}}{\mathrm{TP}+\mathrm{FN}}$$

Where: FN (False negative) is the number of positive samples missed, that is, the target marked with fractures is not detected as fractures.

mAP50 (IoU threshold of 0.5) and mAP_50:95_ (IoU threshold ranging from 0.5 to 0.95) were used as evaluation metrics for mean average precision mAP. The calculation formula of mAP is as follows:


$$\mathrm{mAP}=\frac{1}{N}\sum_{i=1}^{N}AP_{i}$$

Where: *N* is the number of detection target categories, set to 1 in this paper; AP*_i_* is the average precision of category *i*, and its calculation formula is:


$$AP_{i}=\int_{0}^{1}P_{i}R_{i}$$

Where: $P_{i}$ and $R_{i}$ represent the precision and recall of detection category *i* respectively.

## 3 Results

The precision (P), recall (R), mAP50 and mAP50-95 of the YOLO26n-seg and the improved YOLO26n-seg-SAConv models on the dataset of this paper are shown in Table 3. The overall mean average precision (mAP50) of the YOLO26n-seg model in vertebral compression fracture classification is 70.3%, and that of the improved YOLO26n-seg-SAConv model is increased to 74.2%. It can be seen that the improved model has improved performance in vertebral compression fracture classification. Specifically, the mean average precision (mAP50) of the improved model for Type 1, Type 2 and Type 3 is increased by 6.8, −0.2 and 6.3%, respectively, compared with the original model. Although the precision of Type 2 is slightly decreased, the precision of the other two types is greatly improved, which effectively improves the overall precision of vertebral compression fracture classification.

**Table 3:** Comparison of model precision and parameters before and after improvement.

Model		Class	All	Type 1	Type 2	Type 3
YOLO26n-seg		Images	194	117	113	30
Params	FLOPs	Mask(P)	0.666	0.63	0.674	0.696
2.69 M	9.6G	R	0.664	0.605	0.798	0.588
FPS		mAP50	0.703	0.629	0.792	0.689
147		mAP50-95	0.397	0.445	0.519	0.387
YOLO26n-seg-SAConv		Images	194	117	113	30
Params	FLOPs	Mask(P)	0.697	0.722	0.672	0.695
3.53 M	8.0G	R	0.703	0.605	0.787	0.718
FPS		mAP50	0.742	0.696	0.789	0.741
186		mAP50-95	0.481	0.502	0.513	0.429

In addition, the computational complexity (FLOPs) of the improved YOLO26n-seg-SAConv model is reduced by 16.67% compared with the original YOLO26n-seg model. The YOLO26n-seg-SAConv greatly reduces the model computational complexity, and the improved model reduces the demand for computing power, making it more suitable for deployment on edge devices with limited computing power and improving the detection speed under the same computing power.

**Figure 5:**
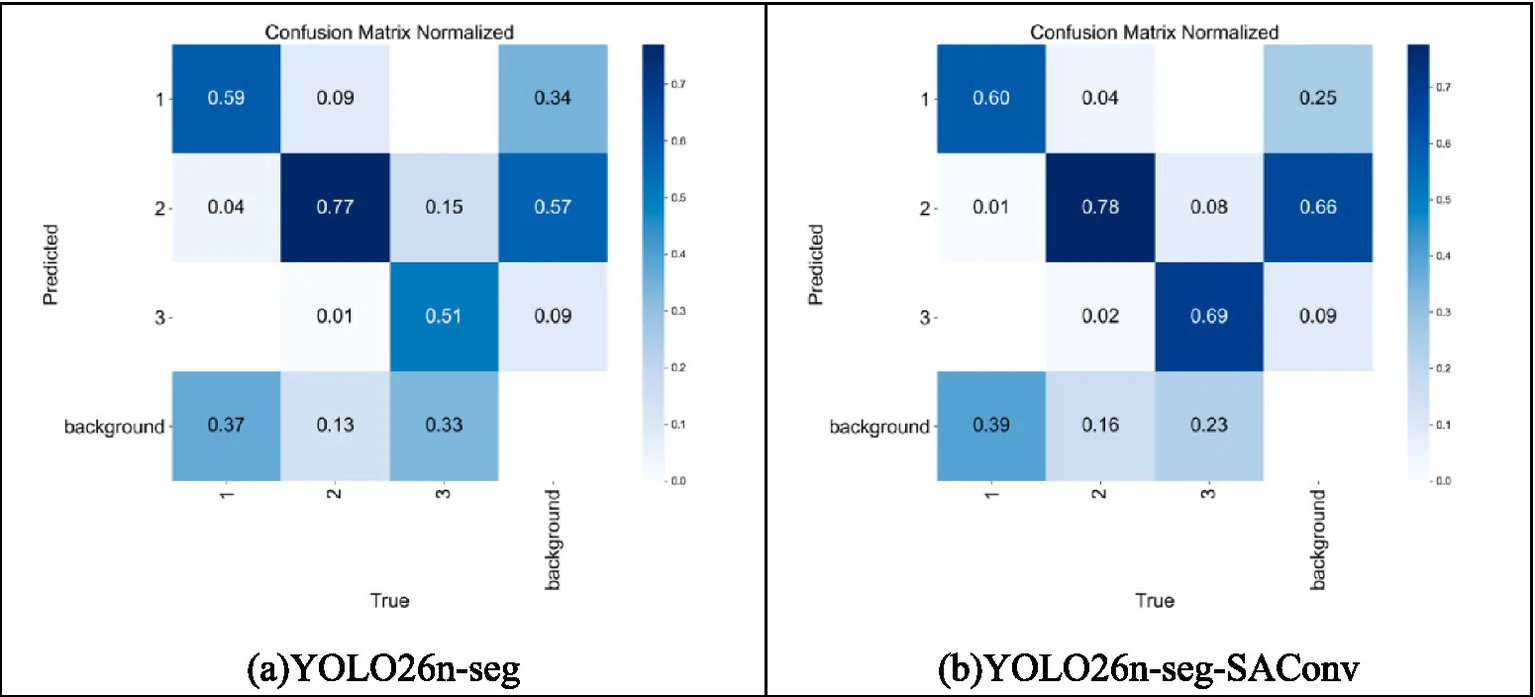
Comparison of normalized confusion matrices of the models. **(a)** shows results obtained by YOLO26n-seg, and **(b)** shows results obtained by YOLO26n-seg-SAConv for visual comparison.

The confusion matrix shown in Figure 5 intuitively displays the classification results of the model for the three types before and after improvement. (a) is the confusion matrix of the original model, and (b) is the confusion matrix of the improved model. The performance of the YOLO26n-seg and the improved YOLO26n-seg-SAConv algorithms were evaluated on the constructed dataset of 966 samples in this paper. Among the three types analyzed, the correct recognition rates of the YOLO26n-seg model for the three types of samples are 59, 77 and 51% respectively, and the detection accuracy of the improved YOLO26n-seg-SAConv for Type 1, Type 2 and Type 3 is increased to 60, 78 and 69% respectively, with the most significant increase in the detection accuracy of Type 3 fractures. This indicates that the SAConv module added in this paper improves the model’s ability to extract features in DR images, and the improvement is the most obvious for Type 3 fractures with a small number of samples, showing that this improvement has a certain effect on the improvement of small sample datasets and the overall detection performance is improved.

**Figure 6:**
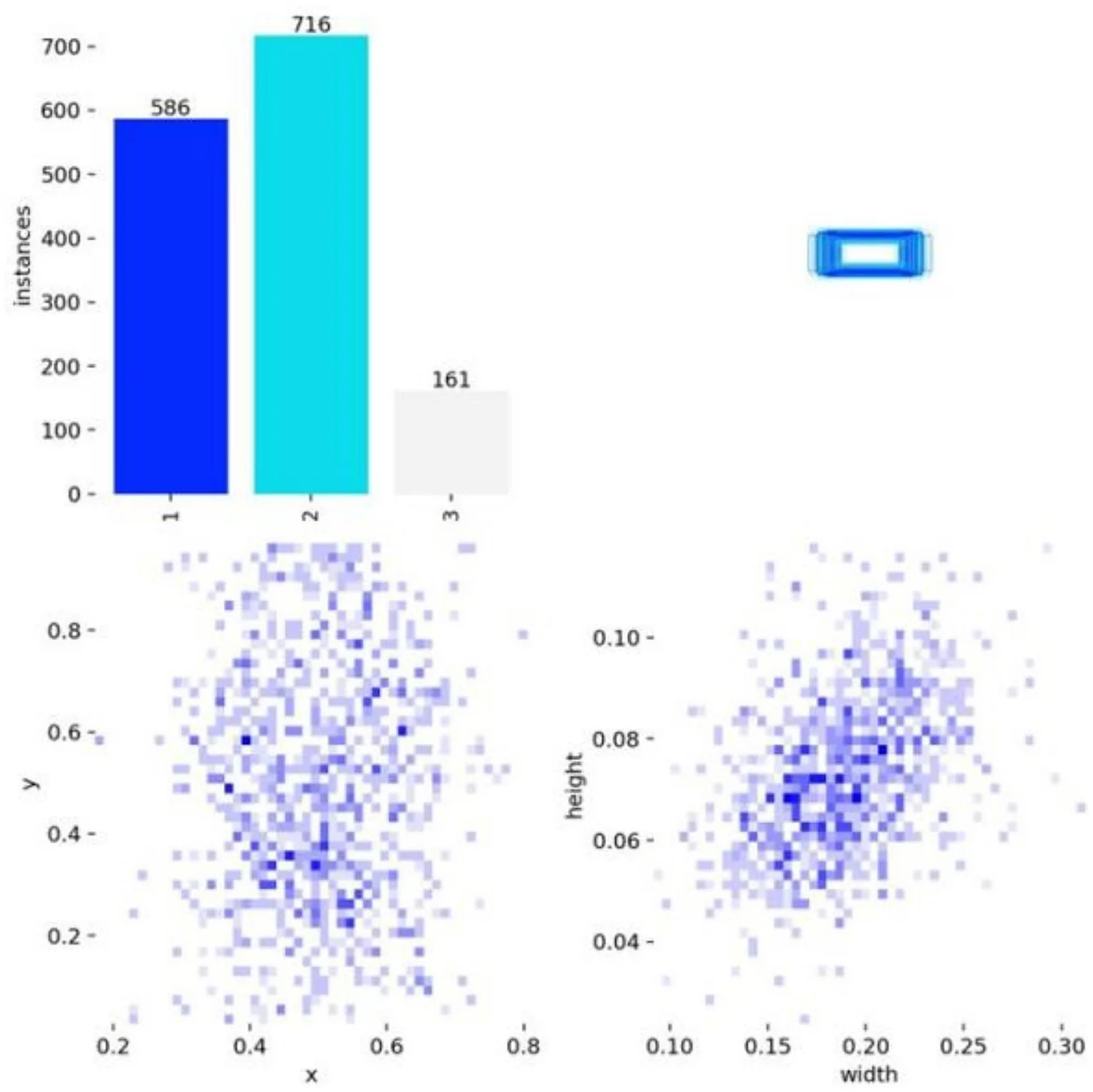
Number and distribution of labels.

Figure 6 shows the data of the training set, including the number of instances in each category, the size and number of target boxes, the position of the center point relative to the entire image, and the aspect ratio of the target in the image. The upper left corner is the number of instances of each category in the training set, that is, how many times the category appears on all training set images. The number of target samples of the three types in the dataset used in this paper is slightly different, which can provide a basis for understanding the number of samples of various defects and data augmentation in the follow-up; the upper right corner shows the effect of aligning and superimposing the detection boxes of all categories with the image center as the origin, which can intuitively understand the shape, size and general position distribution of various bounding boxes; the lower left corner is the position of the center point relative to the entire image, which can further analyze the spatial position preference of defects or targets in the image; the lower right corner is the aspect ratio of the target in the image relative to the entire image, which can further analyze the size and aspect ratio distribution of defect bounding boxes.

**Figure 7:**
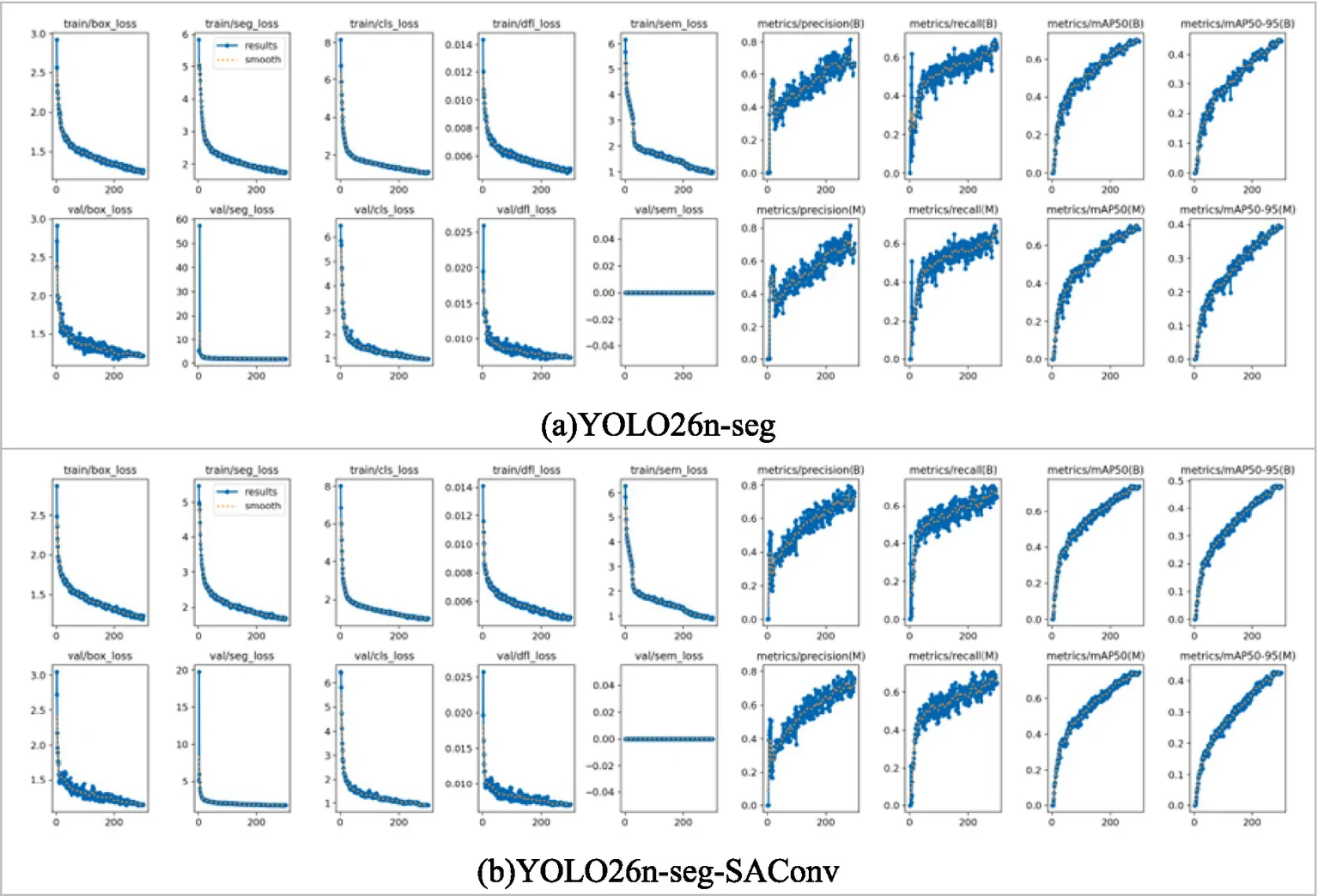
Comparison of model loss curves and precision curves. **(a)** shows results obtained by YOLO26n-seg, and **(b)** shows results obtained by YOLO26n-seg-SAConv for visual comparison.

Figure 7 shows the loss change curve and the change curves of precision, recall and other metrics during the model training process (a) is the curve of the original model, and (b) is the curve of the improved model. The curve showing a downward trend in Figure 7 is the loss change curve. The model evaluates the difference between the predicted value and the actual value through the loss function, which significantly affects the model performance because they guide the training process towards more accurate predictions. The loss function is divided into classification and regression parts: the classification loss function cls_loss (Classification Loss) uses Binary Cross-Entropy Loss to calculate the loss value between the predicted category and the real category. A lower classification loss means that the detected objects are more accurately classified into their respective categories, thus ensuring that the model accurately identifies the types of existing objects. The loss function of the regression branch adopts dfl_loss (Distributed Focal Loss) and box_loss (Bounding box Loss), among which box_loss is used to calculate the loss of the predicted box in terms of position and size compared with the real box, and the error is calculated using the Intersection over Union (IoU) metric. A lower IoU value indicates higher positioning accuracy, reflecting a more accurate overlap between the predicted bounding box and the real bounding box. In general, these loss functions are indispensable for optimizing the YOLO26 model, thus promoting the improvement of detection accuracy and classification accuracy.

The curves showing an upward trend on the right side of Figure 7 are the change curves of precision, recall, mAP50 and mAP50-95 during the model training process respectively, where B and M represent Box and Mask, respectively. Precision is defined as a measure of the accuracy of the model’s positive predictions, calculated as the ratio of correctly identified positive instances (true positives) to all identified positive instances (true positives and false positives). Higher precision indicates that the model makes fewer false positive errors, thus ensuring that most of its positive predictions are accurate. In contrast, Recall represents the proportion of actual positive samples correctly identified by the model, essentially measuring the model’s ability to identify all relevant instances in the dataset, which is the ratio of true positives to the sum of true positives and false negatives. mAP50 and mAP50-95 represent the mean average precision of the model at a confidence threshold of 0.5 and the mean average precision tested at every 0.05 confidence threshold in the range of 0.5–0.95 respectively, reflecting the most real detection performance of the model in different dimensions.

From the loss function and accuracy curves in Figure 7, it can be observed that the loss curve gradually decreases and tends to flatten over the 300 epochs of iterative training, while the accuracy curve rises rapidly in approximately the first 250 epochs and stabilizes after 250 epochs, indicating that the model has reached a fitted state. Moreover, it can be seen from the figure that the accuracy curve of the improved model in (b) is smoother compared to that in (a), and the model reaches the fitted state earlier.

**Figure 8:**
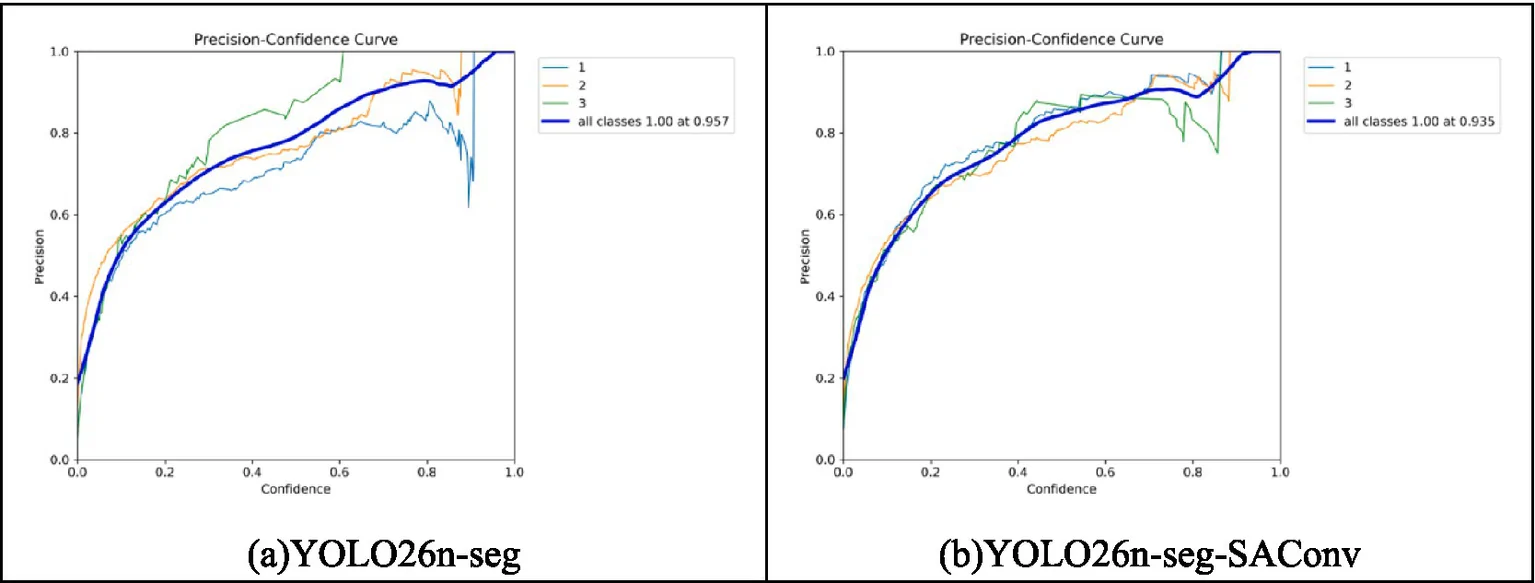
Comparison of Model P curves. **(a)** shows results obtained by YOLO26n-seg, and **(b)** shows results obtained by YOLO26n-seg-SAConv for visual comparison.

Next, the YOLO26n-seg models before and after improvement were compared from four indicators: accuracy, recall, F1 score and AP value, to comprehensively analyze the improvement of their performance in various aspects. First is the accuracy of the three types. In this experiment, accuracy P represents the proportion of the number of types correctly predicted by the model to the total number of types predicted. Figure 8 is the comparison chart of the P curves of the model before and after improvement. (a) is the curve of the original model, and (b) is the curve of the improved model. It can be seen from the figure that the P of the improved model reaches 1 at Confidence = 0.935, which is 0.022 lower than that before improvement, and the accuracy of the improved YOLO26n-seg-SAConv model for all category recognition is slightly improved compared with the original YOLO26n-seg in terms of detection accuracy.

**Figure 9:**
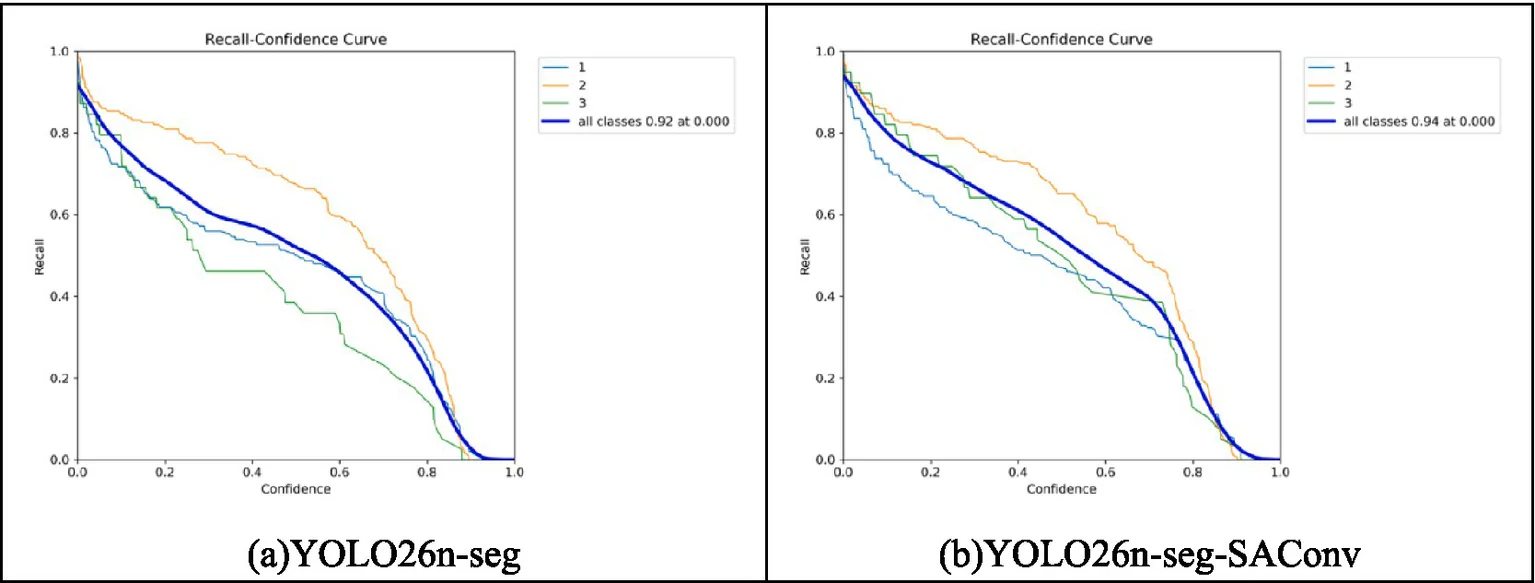
Comparison of model R curves. **(a)** shows results obtained by YOLO26n-seg, and **(b)** shows results obtained by YOLO26n-seg-SAConv for visual comparison.

Recall R represents the proportion of the number of fracture types correctly predicted by the model to the total number of fractures in this experiment. Figure 9 is the comparison chart of the R curves of the model before and after improvement. (a) is the curve of the original model, and (b) is the curve of the improved model. It can be seen from the figure that the R curve scores of the improved model are all 0.94, an increase of 2% compared with 0.92 before improvement, and the R curve of each type is improved compared with that before improvement. Therefore, the improved YOLO26n-seg-SAConv model has a certain improvement in recall.

**Figure 10:**
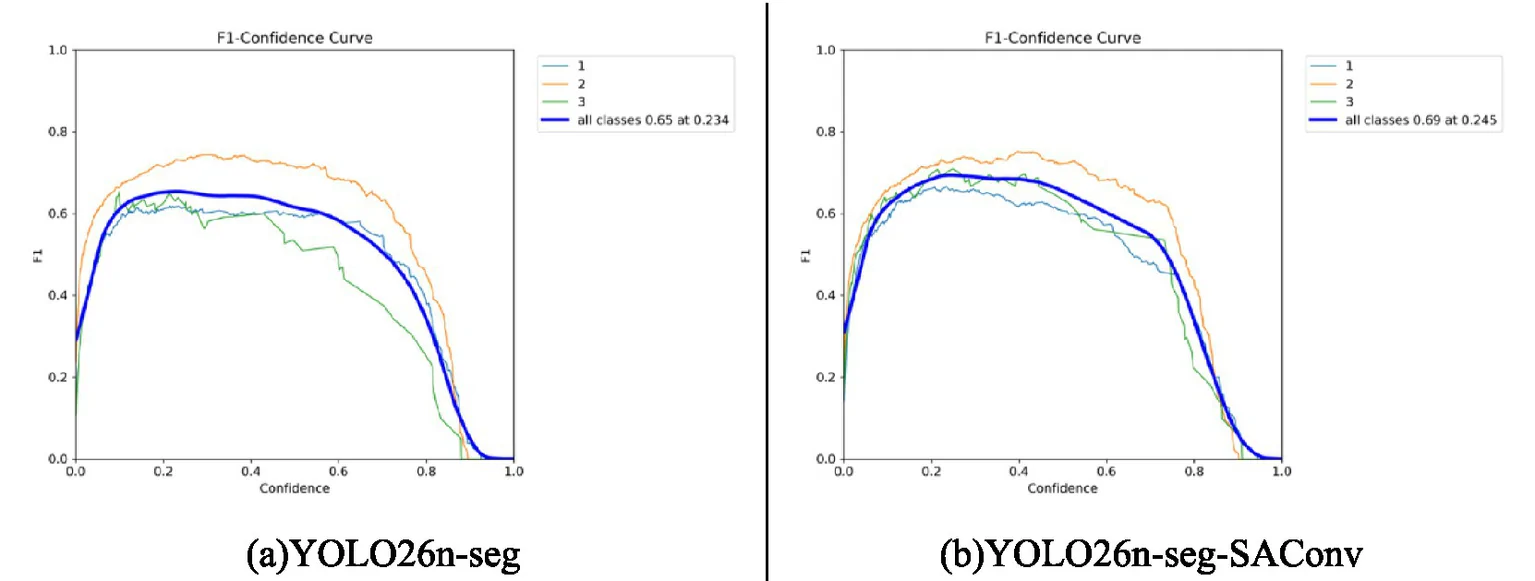
Comparison of Model F1 Curves. **(a)** shows results obtained by YOLO26n-seg, and **(b)** shows results obtained by YOLO26n-seg-SAConv for visual comparison.

The F1 score is the harmonic mean of accuracy P and recall R, which integrates the two indicators. With the threshold set to 0.5, Figure 10 shows the F1 curves of the models before and after improvement. (a) is the curve of the original model, and (b) is the curve of the improved model. It can be seen from the figure that the F1 curves of the improved model for the three different types of targets are all improved compared with those before improvement, and the comprehensive F1 score is increased from 0.65 to 0.69, which improves the F1 score of the model.

**Figure 11:**
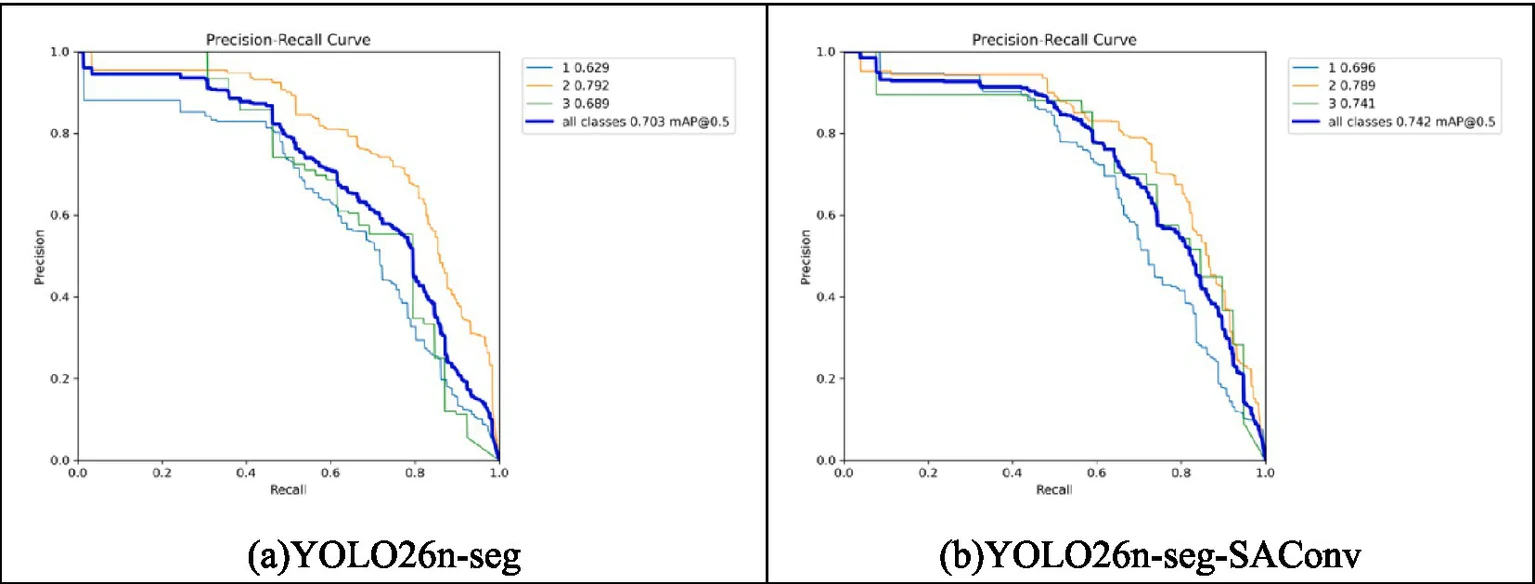
Comparison of model PR curves.

As the most important one among the four indicators, the AP value can more comprehensively measure the relationship between accuracy and recall. In fact, the AP value is the area enclosed by the PR curve and the coordinate axes. A larger area means a larger AP value, that is, the model has a higher detection accuracy for such objects. Figure 11 shows the comparison chart of the PR curves of the model before and after improvement. After replacing the traditional Conv module with the SAConv downsampling module, the changes in the AP values of each type can be seen from the figure. (a) is the curve of the original model, and (b) is the curve of the improved model. Among them, Type 1 is increased from 0.629 to 0.696, Type 2 is slightly decreased from 0.792 to 0.789, and Type 3 is increased from 0.689 to 0.741. On the whole, the mean average precision (mAP) of the three types of fractures is increased by 3.9%, from the original 0.703 to 0.742. After introducing the SAConv downsampling module, the detection performance of the model has been significantly improved.

**Figure 12:**
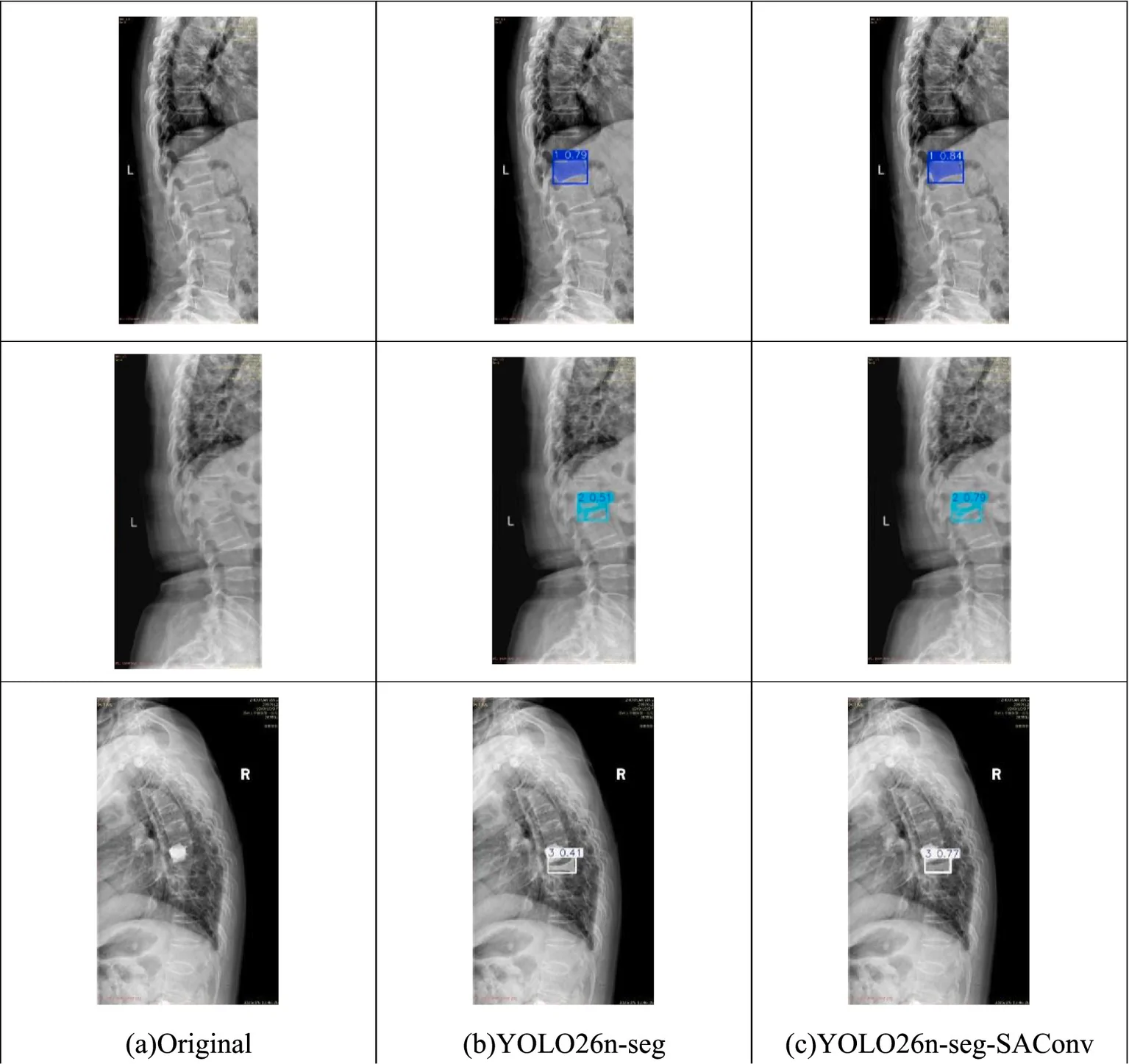
Comparison of visual detection results. **(a)** shows original X-ray image, **(b)** shows results of YOLO26n-seg, and **(c)** shows results of YOLO26n-seg-SAConv.

Figure 12 shows the visual recognition effects of the YOLO26n-seg model and the YOLO26n-seg-SAConv model. (b) is the detection result of the original model, and (c) is the detection result of the improved model. It can be seen from the figure that the improved model has improved the recognition confidence for the three types, with the confidence of Type 1 increased from 0.88 to 0.94, Type 2 from 0.86 to 0.89, and Type 3 from 0.90 to 0.92, which further proves that the improved model has improved the recognition ability for the three types of fractures.

To highlight the performance advantages of the baseline YOLO26n-seg model and to facilitate a horizontal comparison of key parameters across different models, this study incorporates three additional comparative experiments with YOLOv5n-seg, YOLOv8n-seg, and YOLO11n-seg. The experimental results are summarized in the Table 4.

**Table 4:** Comparative experiment.

Model	P	R	mAP50	mAP50-95
YOLOv5n-seg	0.612	0.606	0.645	0.301
YOLOv8n-seg	0.639	0.629	0.678	0.313
YOLO11n-seg	0.653	0.657	0.686	0.318
YOLO26n-seg	0.666	0.664	0.703	0.397

From the comparative experimental results in the table, it can be observed that the YOLO26n-seg model achieves P, R, mAP50, and mAP50-95 values of 0.666, 0.664, 0.703, and 0.397, respectively, demonstrating favorable performance across all four accuracy metrics.

To avoid the experimental results being attributed to chance, a 5-fold cross-validation experiment was conducted to verify the performance improvement before and after adding the SAConv module, thereby making the results more convincing. In the 5-fold cross-validation experiment, the dataset was randomly divided into two equal parts, with one part used as the training set and the other as the validation set. The 5-fold cross-validation results are shown in the table. According to the experimental results in Table 5, it can be concluded that the YOLO26n-seg-SAConv model proposed in this paper achieves consistent improvement when using any half of the fracture detection dataset. After averaging the results of the two groups, the improved YOLO26n-seg-SAConv model achieves improvements of 2.8, 3.4, 2.2, and 1.8% in P, R, mAP50, and mAP50-95, respectively. The performance improvement of the model is stable.

**Table 5:** 5-fold cross-validation experiment.

Group	Model	Mask(P)	R	mAP0.5	mAP0.5:0.95
Group1	YOLO26n-seg	0.644	0.643	0.689	0.316
	YOLO26n-seg-SAConv	0.67	0.676	0.709	0.335
Group2	YOLO26n-seg	0.637	0.638	0.687	0.321
	YOLO26n-seg-SAConv	0.668	0.674	0.711	0.339
Average	YOLO26n-seg	0.641	0.641	0.688	0.319
	YOLO26n-seg-SAConv	0.669	0.675	0.71	0.337

## 4 Discussion

Aiming at the problems of low efficiency in clinical classification and imaging underreporting of osteoporotic vertebral compression fractures (OVCF), this study constructed an improved YOLO26n-seg-SAConv model by introducing the SAConv module based on the YOLO26n-seg model to assist clinicians in performing more efficient OVCF classification (23, 24).

The experimental results of this study show that the improved YOLO26n-seg-SAConv model performs better than the original YOLO26n-seg model in the OVCF classification task, with its overall mean average precision (mAP50) increased from 70.3 to 74.2% and computational complexity (FLOPs) decreased by 16.67%, which fully verifies the effectiveness of introducing the SAConv module. From the perspective of specific classification results, the mAP50 of Type 1 and Type 3 fractures is increased by 6.8 and 6.3% respectively, while the precision of Type 2 fractures only slightly decreases by 0.2%. This difference is closely related to the dataset characteristics and model improvement logic.

The significant improvement in the precision of Type 1 fractures is mainly due to the dynamic multi-scale perception and weight locking mechanism of the SAConv module. Type 1 OVCF in DR images often presents as slight vertebral compression or marginal bone damage, which is a typical small target. The traditional Conv downsampling module is prone to lose such subtle features during the convolution process with a stride of 2, leading to missed detection or misjudgment. The SAConv module, through the design of dual-branch atrous convolution, the low atrous rate branch focuses on the fine depiction of local textures, the high atrous rate branch captures large-scale structural correlations, and then combines the learnable switch function to adaptively fuse the dual-path features, which effectively retains the feature information of small targets and suppresses the interference of background noise in DR images, which is consistent with the advantages of the SAConv module in small target detection tasks (25, 26).

The slight decrease in the precision of Type 2 fractures is mainly due to the bias of model optimization. The SAConv module’s enhancement of subtle features may weaken the capture of the relatively obvious vertebral compression features of Type 2 fractures, resulting in a slight fluctuation in its precision, but this fluctuation is within the clinically acceptable range and does not affect the overall performance of the model.

From the perspective of misdiagnosis and missed diagnosis, the classification errors of the model are mainly concentrated in three typical scenarios. First, missed diagnosis of Type 1 fractures remains the dominant error type. For OVCF cases with extremely mild vertebral compression and no obvious cortical interruption, the subtle morphological changes are easily confused with normal physiological curvature of vertebrae or degenerative changes on DR images. Even with the feature enhancement of the SAConv module, the model still cannot fully capture extremely weak texture features under low image contrast, resulting in false negative results. Second, Genant grade I and grade II fractures are easily confused. The core reason lies in the critical gray zone defined by the 25% vertebral compression cutoff, and the superimposed interference from X-ray projection position errors, inconsistent measurement point selection criteria and atypical fracture morphologies tends to cause cross-type misjudgment. Third, a small number of Type 3 fractures are misdiagnosed as Type 2 fractures. Limited by the sample size of severe fractures, the model has insufficient learning of extreme morphological features of severely compressed vertebrae, and tends to misclassify severe compression fractures with relatively intact vertebral contours into Type 2. In addition, interference factors such as vertebral osteophytes, scoliosis and imaging artifacts in DR images will also interfere with the model’s vertebral edge localization and feature extraction, which is an important inducement for both false positive and false negative results.

In addition, the computational complexity of the improved model is significantly reduced. The Params increased from 2.69 M to 3.53 M but still maintained a lightweight design, and the FLOPs decreased from 9.6G to 8.0G. This advantage makes it more suitable for deployment on clinical edge devices with limited computing power, solving the pain points of traditional deep learning models such as high computing power demand and difficulty in clinical implementation, and providing technical possibilities for the rapid on-site classification of OVCF (27, 28). Visual results such as confusion matrix, P-R curve and F1 curve further confirm that the classification accuracy and recall of the improved model are both improved, especially the correct recognition rate of Type 3 fractures is increased from 51 to 69%, which fully shows that the model’s recognition ability for small sample categories has been significantly improved. This is closely related to the synergistic effect of data augmentation technology (Gaussian noise addition) and the feature extraction advantages of the SAConv module (25).

Current deep learning research on OVCF mostly focuses on the early diagnosis of fractures, that is, judging whether vertebral fractures occur, while there is a lack of research on deep learning models for OVCF classification. As the most widely used OVCF classification method in clinical practice, traditional manual Genant semi-quantitative classification relies on physicians’ experience and has the problems of low efficiency, strong subjectivity and high underreporting rate. The advantages of this study are mainly reflected in three aspects: first, the latest YOLO26n-seg model is selected as the basic framework. As the latest iterative version of the YOLO series, this model natively supports end-to-end inference without NMS post-processing, with faster inference speed. At the same time, it improves the feature extraction ability through modules such as C2PSA and C3K2, and achieves a better balance between lightweight and precision compared with models such as YOLOv8 and YOLO11 (29); second, the SAConv module is innovatively introduced into the model to specifically solve the problem of subtle fracture feature loss in OVCF classification, which is different from the general optimization of existing research and is more in line with the feature characteristics of OVCF in DR images (30); third, a dataset containing 483 DR images covering three Genant classifications is constructed, and data augmentation is realized by adding Gaussian noise to alleviate the small sample problem. At the same time, a class-weighted loss function is used to balance class differences and improve the robustness of the model.

Although this study has achieved certain research results, it still has some limitations that need to be further improved in the follow-up research. First, the scale and diversity of the dataset need to be improved. The dataset of this study only includes 483 DR images from our hospital and affiliated institutions from January 2021 to January 2026, with a relatively single sample source, and the distribution of samples of different age groups and different degrees of osteoporosis is not uniform enough, which may lead to limited generalization ability of the model and difficulty in adapting to the OVCF classification needs of different regions and populations (31–33). Second, there is still room for optimization in model improvement. The hyperparameters of the SAConv module (such as atrous rate and switch function weight) adopt default settings and are not finely tuned according to the characteristics of OVCF DR images, which may affect the model’s adaptability to different types of fractures; at the same time, the model does not consider interference factors such as artifacts and vertebral degeneration in DR images, which may lead to classification errors in some complex cases (25, 34).

In view of the limitations of this study, future research can be carried out from the following aspects: first, expand the scale of the dataset, collect OVCF DR images from different regions, hospitals, age groups and degrees of osteoporosis, and include interference samples such as vertebral degeneration and artifacts to improve the generalization ability of the model (31); second, optimize the model structure, finely tune the hyperparameters of the SAConv module, further improve the model’s classification precision for complex cases combined with attention mechanism and feature fusion technology, and try to introduce multi-modal data such as CT and MRI images to make up for the information shortage of DR images (35, 36); third, carry out multi-center clinical controlled trials, conduct blind comparison with the manual classification of clinicians, verify the clinical practicability and reliability of the model, and collect clinical feedback to optimize the adaptability of the model (11, 37); fourth, promote the clinical translation of the model to transform methodological validation outcomes into practical auxiliary diagnostic tools for routine clinical workflows.

## 5 Conclusion

By introducing the SAConv module into the YOLO26n-seg framework, this study develops the YOLO26n-seg-SAConv model for automated and rapid classification of OVCF. As a methodological validation work, it provides a feasible technical solution to the low efficiency of traditional manual classification and the high computational demand of existing deep learning models. Experimental results demonstrate that the improved model reaches an overall mAP50 of 74.2% with a 16.67% reduction in computational complexity. Specifically, the recognition performance for Type 1 and Type 3 fractures is significantly improved, and its lightweight design lays a technical foundation for deployment on clinical edge devices.

Although the study has limitations including a single-center dataset and absence of clinical controlled trials, the proposed model provides new technical support for algorithmic research on automated OVCF classification, with notable methodological value and clinical translational potential. In future work, the performance and clinical applicability of the model can be further enhanced through dataset expansion, structural optimization and clinical validation, advancing the automation, precision and lightweight implementation of OVCF imaging evaluation.

## Data Availability

The raw data supporting the conclusions of this article will be made available by the authors, without undue reservation.
